# The Fusion Protein Signal-Peptide-Coding Region of Canine Distemper Virus: A Useful Tool for Phylogenetic Reconstruction and Lineage Identification

**DOI:** 10.1371/journal.pone.0063595

**Published:** 2013-05-13

**Authors:** Nicolás Sarute, Marina Gallo Calderón, Ruben Pérez, José La Torre, Martín Hernández, Lourdes Francia, Yanina Panzera

**Affiliations:** 1 Sección Genética Evolutiva, Instituto de Biología, Facultad de Ciencias, Universidad de la República, Montevideo, Uruguay; 2 Centro de Virología Animal, Instituto de Ciencia y Tecnología Dr. Cesar Milstein, Buenos Aires, Argentina; Radboud University Medical Centre, NCMLS, The Netherlands

## Abstract

Canine distemper virus (CDV; *Paramyxoviridae, Morbillivirus*) is the etiologic agent of a multisystemic infectious disease affecting all terrestrial carnivore families with high incidence and mortality in domestic dogs. Sequence analysis of the hemagglutinin (H) gene has been widely employed to characterize field strains, permitting the identification of nine CDV lineages worldwide. Recently, it has been established that the sequences of the fusion protein signal-peptide (Fsp) coding region are extremely variable, suggesting that analysis of its sequence might be useful for strain characterization studies. However, the divergence of Fsp sequences among worldwide strains and its phylogenetic resolution has not yet been evaluated. We constructed datasets containing the Fsp-coding region and H gene sequences of the same strains belonging to eight CDV lineages. Both datasets were used to evaluate their phylogenetic resolution. The phylogenetic analysis revealed that both datasets clustered the same strains into eight different branches, corresponding to CDV lineages. The inter-lineage amino acid divergence was fourfold greater for the Fsp peptide than for the H protein. The likelihood mapping revealed that both datasets display strong phylogenetic signals in the region of well-resolved topologies. These features indicate that Fsp-coding region sequence analysis is suitable for evolutionary studies as it allows for straightforward identification of CDV lineages.

## Introduction

Canine distemper virus (CDV) is a member of the *Morbillivirus* genus within the *Paramyxoviridae* family. The viral genome consists of a nonsegmented single-stranded negative RNA of 15.7 kb and encodes six structural proteins. The nucleocapsid protein, viral polymerase, and phosphoprotein are associated with the genomic RNA forming the ribonucleoprotein complex, which performs viral transcription and replication. The hemagglutinin (H) and fusion (F) proteins are the antigenic determinants of the virus and are located at the viral envelope, whereas the matrix protein is membrane associated [1].

CDV is the etiologic agent of canine distemper (CD), a severe multisystemic infectious disease, which is distributed worldwide and affects all terrestrial carnivores [2]–[4]. CD has been controlled by attenuated vaccines; however, in recent decades, several outbreaks in properly vaccinated dogs and an expansion in the host range have been reported [5]–[11]. These outbreaks might be a consequence of the emergence of new field strains able to avoid the immune response generated by the “old strains” currently used in the vaccines and/or because of the capacity of new field strains to infect other carnivore hosts [12]–[20].

The H gene shows the greatest genetic variation within the *Morbillivirus* genus, and it has been widely employed to characterize field strains [21]. In CDV, the amino acid divergence of the H protein reaches 10% among field strains [12], [22]. This variability in conjunction with a phylogenetic analysis provide the necessary information for the classification of CDV strains into genetic lineages: two strains belong to the same lineage if they cluster together and share an amino acid divergence of <3.5%; the strains belong to different lineages if they appear in separate clades and show values of divergence >4% [12], [22]. Currently, nine lineages have been characterized worldwide according mainly to their geographic origin [9], [22]–[25]. In South America there are two co-circulating lineages, the South America 2 (SA2) lineage exclusive to South America and the Europe 1/South America 1 (EU1/SA1) lineage, which is spread in Europe and South America [25].

The H gene can be more difficult to amplify directly from field samples because of its larger size (1824 bp) [17], [23]–[25], and its transcription level is proportionately lower in comparison to the genes located in the 3′ terminal region of the genome [1].

It has been recently established that a short region of the F gene that encodes the signal peptide of the F protein (Fsp; residues 1–135) is extremely variable. Comparative analysis of the Fsp peptide from Asian strains shows that it has substantial genetic variability, suggesting that this region could be a marker for classifying CDV strains [26], [27].

The aim of this work was to evaluate the phylogenetic resolution of the Fsp-coding region in comparison with the H gene. The analyses revealed that the Fsp-coding region is phylogenetically informative and quite useful for the identification of genetic CDV lineages, which will allow for rapid characterization of circulating strains.

## Materials and Methods

### Datasets

To analyze the phylogenetic resolution of the Fsp-coding region and compare it with that of the H gene, we constructed datasets for both genomic regions from the same 37 strains (Table 1). The H dataset included strains available at GenBank for eight of the nine CDV lineages; sequences from the African lineage strains were not included because there are no records of the Fsp-coding region sequences in these strains. The Fsp dataset included 29 sequences available at GenBank and eight sequences obtained here from the EU1/SA1 and SA2 lineages (Table 1).

**Table 1:** Strains employed for the construction of the Fsp and H datasets.

Strain	Fsp region	H gene	Lineage/Clade
UY102	KC331150	JN215473	EU1/SA1
UY111	KC331151	JN215475	EU1/SA1
UY128	KC331152	JN215476	EU1/SA1
UY141	KC331153	JN215477	EU1/SA1
Arg23	KC257465	FJ392652	EU1/SA1
5804	AY386315	AY386315	EU1/SA1
5804P	AY386316	AY386316	EU1/SA1
Arg24	KC257466	FJ392651	SA2
Arg25	KC257467	KC257463	SA2
Arg26	KC257468	KC257464	SA2
MS01	EF445055	DQ922630	AS1
JL(07)1	EU327875	EU325728	AS1
HeB(07)1	EU327874	EU325720	AS1
BS0610	EU934234	EU934233	AS1
GS0812-4	HQ850148	HQ850147	AS1
GN	EF596900	EF445054	AS1
SC01	EF596902	EF042818	AS1
ZD01	EF596904	EF445051	AS1
CDV GZ2	JN381189	JN381191	AS1
NM	EF596903	EF445053	AS1
W812B	AB607905	AB605890	AS1
W729B	AB607904	AB605891	AS1
007Lm	AB474397	AB474397	AS2
007Lm-1vp	AB462810	AB462810	AS2
19876	AY964110	AY964110	EU2
18133	AY964108	AY964108	EU3
HL	EF596901	EF445052	EU3
21261	AY964112	AY964112	EU3
25259	AY964114	AY964114	EU3
A75/17	AF164967	AF164967	NA1
164071	EU716337	EU716337	NA1
98-2654	AY466011	AY466011	NA2
98-2645	AY445077	AY445077	NA2
98-2646	AY542312	AY542312	NA2
Onderstepoort (OS)	AF378705	AF378705	NA2
Snyder Hill	GU138403	GU138403	NA2
Onderstepoort (97)	AF014953	AF014953	NA2

Denomination, GenBank accession number for the Fsp-coding region and H gene, and lineage for each strain are detailed. AS1, Asia 1; AS2, Asia 2; EU1/SA1, Europe 1/South America 1; EU2, Europe 2; EU3, Europe 3; NA1, North America 1; NA2, North America 2; SA2, South America 2.

### Amplification of the Fsp-coding Region

Urine, ocular discharge, and clotted blood samples from dogs were subjected to Trizol RNA isolation (Invitrogen) to isolate CDV RNA (Table 1). The RT-PCR products of 681 bp encompassing the Fsp-coding region (405 bp) were amplified using the SuperScript One-Step RT-PCR kit reagents (Invitrogen) (the cycling conditions are available from the authors on request) through a set of newly designed primers, F4854∶5′-TCCAGGACATAGCAAGCCAACA-3′and R5535∶5′-GGTTGATTGGTTCGAGGACTGAA-3′ (strain N° AF378705 position numbering). The PCR products were sequenced bidirectionally on an ABI3130 automated sequencer (Applied Biosystems), and nucleotide sequences were submitted to the GenBank database (http://www.ncbi.nlm.nih.gov).

### Comparative Analyses

To evaluate the phylogenetic resolution of the Fsp dataset and compare it with the H dataset, two bioinformatic approaches were employed: phylogenetic and likelihood mapping (LM) analyses.

### Phylogenetic Analysis

The phylogenetic relationships among the strains were established for both datasets by maximum-likelihood trees using MEGA 5 software [28]. The substitution model used was Hasegawa-Kishino-Yano with gamma distribution (G) for both datasets.

Internal-node uncertainties were assessed using 500 bootstrap replications. Amino acid p-distance analysis was implemented for both datasets with MEGA 5 software [28].

### LM Analysis

The LM method was implemented for both datasets using TREE-PUZZLE software [29]. LM assesses if a dataset is suitable for phylogenetic reconstruction by the analysis of groups of four randomly chosen sequences (quartets). For each quartet, three unrooted tree topologies are possible. The phylogenetic signals are computed as probabilities that are represented in a triangle surface [30].

## Results

### Amplification of the Fsp-coding Region

The 681-bp amplicon that encompasses the 405-bp Fsp-coding region was obtained directly from samples of different origin. The sequences of the eight strains from South American lineages (EU1/SA1 and SA2) were added to the Fsp dataset. The Fsp and H datasets each comprised 37 sequences of strains from eight of the nine CDV lineages characterized (Table 1) and were used to perform the subsequent analyses.

### Comparative Analyses: Phylogenetic and LM Analysis

**Figure 1:**
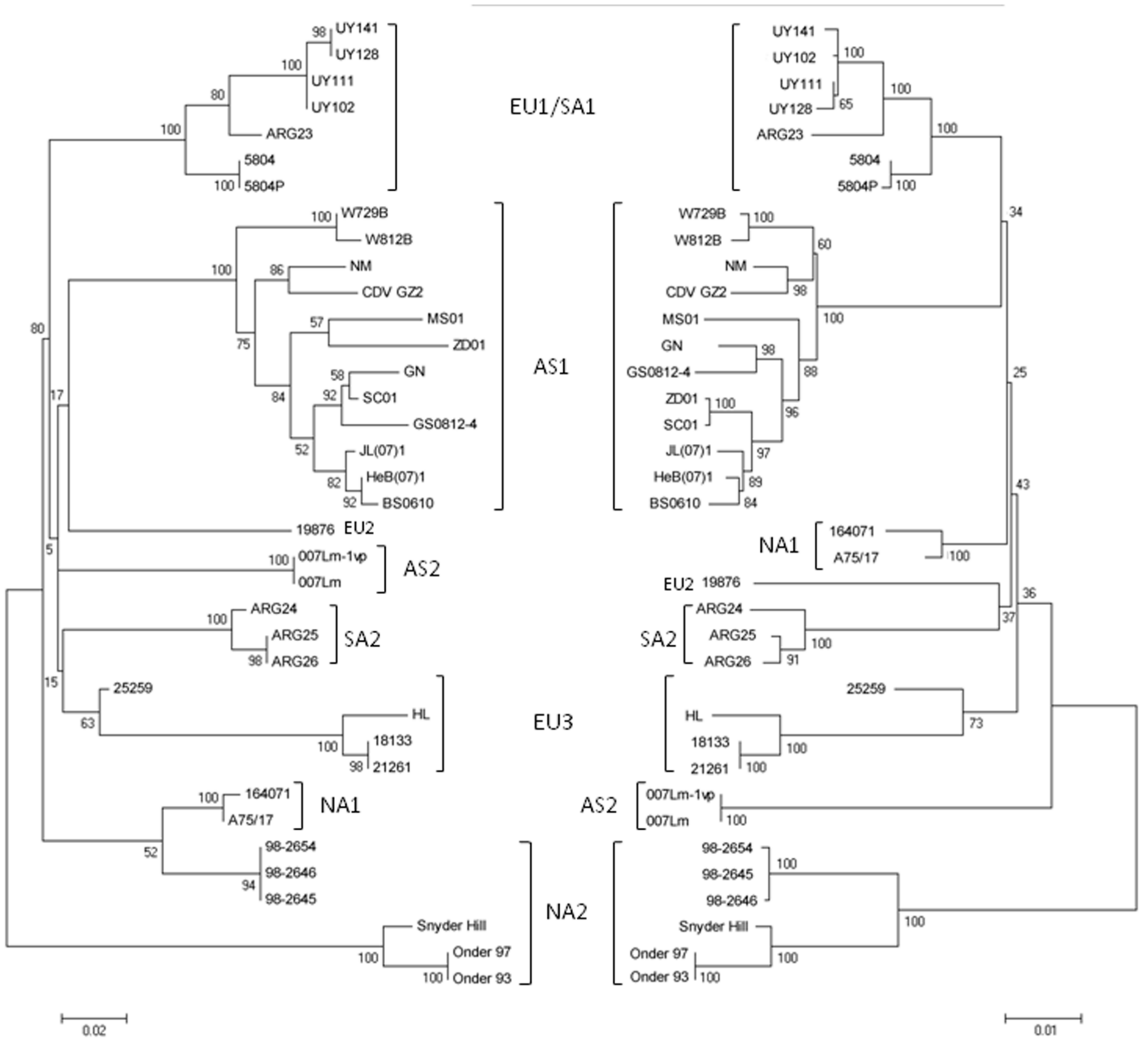
Phylogenetic analysis of CDV isolates. Thirty-seven nucleotide sequences of the Fsp (left) and H (right) datasets were used. Maximum likelihood trees were constructed using the Hasegawa-Kishino-Yano (G) substitution model for both datasets and were inferred through 500 replicates. Branch lengths are measured in the number of substitutions per site, as shown by the scale bars. Unrooted trees were depicted facing each other for comparison. AS1, Asia 1; AS2, Asia 2; EU1/SA1, Europe 1/South America 1; EU2, Europe 2; EU3, Europe 3; NA1, North America 1; NA2, North America 2; SA2, South America 2; Onder, Onderstepoort strain; Snyder, Snyder-Hill strain.

Phylogenetic analysis of the Fsp and H datasets was performed to compare the relationships among the CDV strains. The Fsp and H trees clustered the same strains corresponding to eight CDV lineages with similar bootstrap values. The branch lengths were measured according to the number of substitutions per site for the substitution model (0.01 for both datasets) (Figure 1). In both trees, similar relationships were observed among most lineages. The positions of the North American lineages (NA1 and NA2) were quite different between the trees: the NA1 and NA2 lineages clustered in the same clade in the Fsp tree, whereas these lineages were not related in the H tree (Figure 1). Inter-lineage amino acid divergence (p-distance) for the Fsp dataset ranged from 19.6% to 36.9%, whereas for the H dataset it ranged from 3.4% to 9.9% (Table 2). The relationships among strains within each CDV lineage were slightly different for the two trees. Intra-lineage amino acid divergence was from 1.8% to 20.7% for the Fsp dataset and from 1.3% to 6.3% for the H dataset (Table 2). The EU2 lineage was not considered because it was represented by a single strain.

**Table 2:** Inter- and intra-lineage amino acid divergences (p-distances).

EU1/SA1	11.9								
	3.3								
SA2	23.5	1.8							
	4.7	2.2							
EU2	25	25.8	–						
	4.8	5.7	–						
EU3	29.5	31.1	30.5	18.5					
	5.9	6.8	6.2	4.6					
NA1	19.6	22.6	23.2	27.3	–				
	3.4	4.6	4.2	5	1.3				
NA2	33.6	33.8	31.1	31.9	23.6	20.7			
	8.6	9.8	9.2	9.6	8.4	6.3			
AS1	26.6	27.3	32.5	36.9	25.6	27.8	18.5		
	5.5	6.9	5.9	6.8	4.7	8.7	3.1		
AS2	24.6	28.4	31	26.5	23.2	22.2	33.6	–	
	6.9	8.2	6.9	7.3	6.1	9.9	6.9	–	
	EU1/SA1	SA2	EU2	EU3	NA1	NA2	AS1	AS2	

The upper values represent the divergence for Fsp; the lower values represent the divergence for the H protein. AS1, Asia 1; AS2, Asia 2; EU1/SA1, Europe 1/South America 1; EU2, Europe 2; EU3, Europe 3; NA1, North America 1; NA2, North America 2; SA2, South America 2.

**Figure 2:**
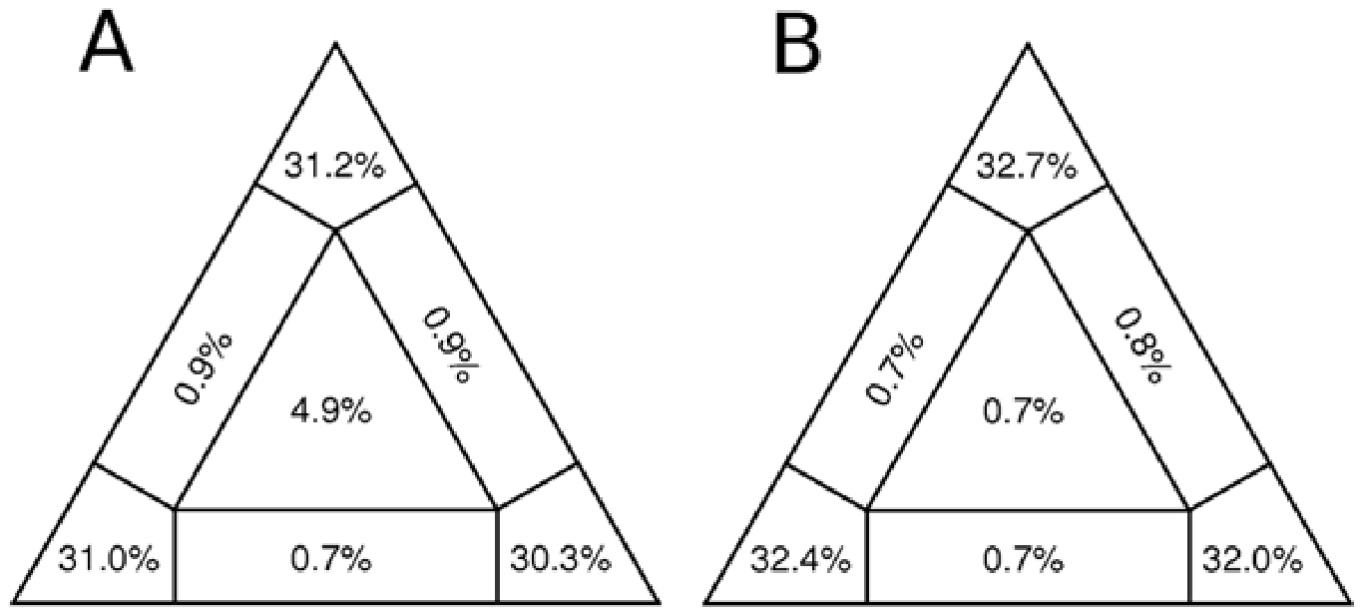
Likelihood mapping of the Fsp (A) and H (B) datasets. The probabilities close to the triangle corners represent tree-like topologies (well-resolved). Those in the center and on the sides represent star-like (unresolved) and network-like signals (partially unresolved), respectively.

The LM analysis showed that the Fsp and H datasets presented probabilities of 92.5% and 97.1%, respectively, in the three corners of the triangle, which represented the tree-like topologies (well-resolved phylogenies). The probabilities in the center region, representing the star-like topologies (unresolved phylogenies), were 4.9% for the Fsp dataset and 0.7% for the H dataset. The probabilities at the sides, representing the net-like region (partially unresolved phylogenies), were 1.8% and 1.5% for the Fsp and H datasets, respectively (Figure 2).

## Discussion

The worldwide spread of canine distemper in all terrestrial carnivore families and some marine carnivores has lead to several studies of this disease in recent decades [4], [5], [6], [11], [18]. Most of the characterization studies of CDV strains have been based on H gene analysis. The direct amplification of the H gene from field samples is difficult without prior propagation of the virus in cell culture [9], [12], [13], [15], [31], [32]. Moreover, its extension (1824 bp) requires the use of several primers sets for amplification and sequencing of the full-length gene, which limits the rapid characterization of CDV strains. The genetic diversity of the Fsp-coding region in Asian strains [26], [27] encouraged us to evaluate the robustness of this region for phylogenetic analysis of the CDV lineages distributed worldwide.

For this work, we constructed Fsp and H datasets and analyzed their phylogenetic resolution. To include the South American strains, we designed a new set of primers, obtaining for the first time, the Fsp-coding region sequences of eight samples belonging to the EU1/SA1 and the SA2 lineages (Table 1). The Fsp-coding region consists of only 405 bp, making it easy to amplify directly from field samples of different origin using conventional methods for RNA isolation.

Phylogenetic analysis performed for the Fsp dataset clustered the same strains into eight well-defined clades according to the CDV lineages based on the H gene (Figure 1). The Fsp amino acid inter-lineage divergence was about fourfold greater with respect to the H protein. This result confirms previous reports regarding Asian strains [26], [27] and also allows us to extend the analysis to the rest of the CDV lineages worldwide, except for the African lineage.

The relationships among the lineages revealed slight differences in the Fsp and the H trees. The positions of the North American lineages (NA1 and NA2) were notably different between the trees: the Fsp tree showed that the NA1 and NA2 lineages were related, whereas the H tree did not show any relationship between them. The Fsp tree might reflect the actual relationships among the North American strains according to the geographical pattern of CDV lineage distribution [9], [12], [22]–[25] (Figure 1). Moreover, the NA1 lineage includes only field strains, whereas the NA2 lineage clusters field and vaccine strains into a main clade in both trees. The field and vaccine strains of the NA2 lineage presented amino acid divergence of up to 6.3% for the H protein, being this value higher than the 4% currently accepted to define lineages [22]. The NA2 main clade would then correspond to two different lineages: the vaccine cluster formed by “old” CDV strains isolated in 1930–1950 and the current field strain clade. For Fsp, the NA2 intra-lineage divergence is 20.7%, which represents the greatest value among all lineages analyzed (Table 2). This might be attributed to the clustering of the field and vaccine strains into this clade, which includes two different lineages (Table 2).

The intra-lineage divergence of Fsp was greater than that of the H protein for most of the lineages, except for the SA2 and NA1 lineages (Table 2). The SA2 lineage is composed of three strains isolated from the same geographical area (Buenos Aires, Argentina), which may have influenced their genomic homogeneity (Table 2). The NA1 lineage includes only two strains A75/17 and 164071, which showed two nucleotide synonymous changes for the Fsp-coding region, and 15 synonymous and two non-synonymous changes for the H gene. Thus, it is necessary to analyze more strains to establish if the variability of the Fsp-coding region is greater than that detected for the H gene as was observed for most of the lineages.

Despite the widespread use of the H gene for phylogenetic analysis of CDV and other *Paramyxovirus* [33]–[35], its phylogenetic signal has not been weighted before. Herein, we applied LM analysis, which revealed a strong phylogenetic signal for the H dataset in the region of tree-like topologies (Figure 2). Thus, this genomic region is phylogenetically informative. The LM analysis for the Fsp dataset showed values >90% in the region of tree-like topologies, indicating that this genomic region is also phylogenetically informative (Figure 2). We propose that the Fsp-coding region could be used as an alternative genetic marker for evolutionary studies and the rapid identification of CDV lineages because of its easy amplification, higher variability, and phylogenetic robustness. The current criteria take into account the H protein amino acid divergence and the clustering of the strains [12], [22]. Based on the Fsp analysis, we established that two strains belong to the same lineage if they cluster in the same clade with values of amino acid divergence <19%, whereas they belong to different lineages if they cluster in different clades with divergence >19%.

In conclusion, we have shown the suitability of the Fsp-coding region for phylogenetic analysis and CDV molecular characterization, which leads us to suggest this genomic region as an alternative marker to identify CDV lineages. The wide employment of the Fsp-coding region will improve the rapid and unequivocal classification of the circulating CDV strains and provide complementary information to understand CDV epidemiology.
